# Project Initiate: A Clinical Feasibility Trial of Equitable Access to Early Neurodevelopmental Therapy

**DOI:** 10.3390/jcm13247681

**Published:** 2024-12-17

**Authors:** Jessica Trenkle, Alison Liddle, Lynn Boswell, Dawn Drumm, Denise Barnes, Aneta M. Jedraszko, Bree Andrews, Shannon Murphy, Michael E. Msall, Deborah Gaebler-Spira, Raye-Ann deRegnier

**Affiliations:** 1 Ann & Robert H. Lurie Children’s Hospital of Chicago, Chicago, IL 60611, USA; 2 Independent Researcher—M Street Pediatric Therapy, Chicago, IL 60647, USA; 3 Department of Pediatrics, Comer Children’s Hospital, University of Chicago, Chicago, IL 60637, USA; 4 Department of Pediatrics, University of Illinois at Chicago, Chicago, IL 60612, USA; 5 Kennedy Research Center on Intellectual and Neurodevelopmental Disabilities, University of Chicago, Chicago, IL 60637, USA; 6 Department of Pediatrics, Northwestern University Feinberg School of Medicine, Chicago, IL 60611, USA; 7 Department of Physical Medicine and Rehabilitation, Northwestern University Feinberg School of Medicine, Chicago, IL 60611, USA; 8 Shirley Ryan AbilityLab, Chicago, IL 60611, USA

**Keywords:** physical therapy, early intervention, service delays, at-risk infant

## Abstract

**Background/Objectives:** Despite evidence of the effectiveness of early intervention (EI) programs, eligible infants often experience delays in initiation of services or fail to receive services entirely. Disparities have been documented, including lower enrollment rates for infants with public insurance. The objective of this pilot study was to evaluate the feasibility of initiating home physical therapy (PT) services promptly after neonatal or cardiac intensive care unit (NICU/CICU) discharge for infants with public insurance and to assess early motor outcomes for children who received study therapy compared with a standard of care group. **Methods:** Infants were recruited if discharged from a study NICU/CICU, had public insurance, and were eligible for Illinois EI services. Infants living in Chicago (*n* = 46) received weekly home-based PT from a study therapist until 3–4 months corrected age (CA). Infants living outside Chicago received standard of care services and served as a control group (*n* = 14). At discharge, infants were referred to EI and underwent the Test of Infant Motor Performance (TIMP). Outcomes at 3–4 months CA included initiation rates for study PT and EI and follow-up TIMP testing. **Results:** By 3–4 months CA, 78% of the intervention group had received ≥1 PT session. In contrast, just 13% of the entire cohort had received any EI therapy. Infants who had 8–10 PT sessions in the first 3–4 months after discharge were more likely to have a change in the TIMP Z-score of >0.5 SD. **Conclusions:** Prompt transition to home therapy was feasible for infants with public insurance in an urban setting who may benefit most due to the potential for neuroplastic change. Addressing barriers identified in this study may assist in improving access to EI for young infants.

## 1. Introduction

Advances in neonatal care have improved the survival of critically ill infants [1,2]. Despite progress, high rates of adverse neurodevelopmental outcomes [2,3,4,5] are reported among infants with diverse neonatal risk conditions, including prematurity, neurologic injuries, and congenital heart disease. Research on neuroplasticity and alterations in developmental trajectories of the brain during neonatal hospitalization has supported the provision of rehabilitation services as a recommended standard of care in neonatal intensive care units (NICUs) and cardiac intensive care units (CICUs) [6,7,8,9]. As a child nears hospital discharge, it is necessary to transition from hospital-based therapy services to home-based services. Home-based services should be available to all qualifying infants through their state’s Part C Early Intervention (EI) program.

All states vary in their eligibility criteria but are required to include infants and toddlers with physical or mental conditions associated with a high probability of developmental delays [10]. Despite evidence of the effectiveness of early therapy services [11,12,13], there are barriers to enrollment. Eligible infants may never receive services, or the initiation of services may be significantly delayed [14,15,16,17]. Although the process of enrollment is the same for all children, disparities with lower enrollment rates and delays in providing services have been described for infants with public insurance, infants with Black or Hispanic parents, families who do not speak English, and younger infants [14,15,16,17,18,19,20].

When there is a delay in initiating therapy services through EI, families with private insurance can access outpatient therapy as a bridge to EI. In contrast, children with public insurance or those living in under-resourced areas often face obstacles in obtaining outpatient therapy. One recent study demonstrated that <10% of outpatient physical therapy (PT) providers in Illinois accepted public insurance as a form of payment [21]. A lack of outpatient options for children with public insurance creates an additional disparity for these families, who often wait many months until services are provided through the EI system. Unfortunately, this also means these infants may miss opportunities to enhance progress during the period of greatest neuroplastic potential.

The objective of this project was to pilot a NICU-to-home service delivery model, “Project INITIATE”, for publicly insured NICU/CICU graduates with risk factors for neuromotor delays and impairments in early childhood. We aimed to evaluate the feasibility of providing home PT services promptly after NICU/CICU discharge as a bridge to initiation of EI. We also aimed to assess early motor outcomes and parental quality of life measures for children who received study therapy compared with infants meeting study eligibility criteria who received standard of care referrals to EI (the control group). The secondary aim of the project was to compare timeliness to EI services for all participants as well as early developmental trajectories.

## 2. Materials and Methods

The pilot study was approved by the Institutional Review Boards at all participating sites, including Ann & Robert H. Lurie Children’s Hospital of Chicago (IRB2021-4541), Northwestern University (STU00217005), the University of Illinois at Chicago (STUDY2022-0991), and University of Chicago Medicine: Comer Children’s Hospital (IRB22-0437). Written informed consent was obtained from the parents or legal guardians of all participants prior to enrollment. The trial was registered with ClinicalTrials.gov (Identifier: NCT05251051).

Infants were recruited from three urban Level III or Level IV NICUs and one CICU between 1 January 2022 and 28 February 2024. Eligibility criteria included: (1) planned discharge date by 45 weeks postmenstrual age, (2) referral to the hospital’s developmental follow-up clinic, (3) public insurance as primary coverage, (4) presence of at least one of the EI-eligible, high-risk medical diagnoses accepted in Illinois (URL: https://www.dhs.state.il.us/page.aspx?item=96962, accessed on 11 December 2024), and (5) at least one English-speaking parent. Infants meeting study criteria were recruited near the time of hospital discharge. They were assigned to the intervention group if their parents resided in the city of Chicago and to the standard of care group if they lived in a suburb of Chicago. Group assignments were based on the availability of the study PTs to travel to the child’s home, given that the city of Chicago covers 232 square miles.

All infants in this study qualified for EI services based on their neonatal diagnoses according to the state of Illinois eligibility requirements. All infants were referred to EI at discharge by the study team utilizing the standard EI referral form that included the neonatal diagnoses that qualified the infants for EI. Hospital staff also referred nearly all of the patients, but details of the hospital referrals were not uniformly available to the study staff. The duration of this study was chosen to provide “bridge” PT services from discharge until EI services were initiated. By law, after the initial contact with EI, 45 days are allowed for the evaluation, determination of services, and family meeting to develop an individualized Family Service Plan (IFSP) (URL: https://www.ilga.gov/legislation/ilcs/ilcs3.asp?ActID=1463&ChapterID=32, accessed 11 December 2024). Services should begin as soon as possible after the IFSP meeting. Based on this timeline and our aim to identify potential benefits of transition to home services, the study endpoint occurred at the time of the 3–4 months corrected age (CA) assessments. Infants who missed these appointments were assigned a study end date on the date of the missed appointment.

### 2.1. Procedures

The intervention and standard of care groups were supported and monitored by a navigator, who assisted parents with EI enrollment questions and helped to identify options for local therapy as needed (PT, occupational therapy, or speech therapy) as a bridge to EI. The study navigator also elicited parent reports of the timing of each step in the process for obtaining EI services (initial contact with EI, home evaluation, determination of eligibility, and the initiation of EI home therapy services).

For the infants assigned to the intervention group, a connection call was conducted virtually or by telephone between parents, the study coordinator, and the study therapist around the time of hospital discharge. When possible, the hospital therapist was also included. The connection calls served to discuss the goals and objectives of PT for their infants, review the PT interventions their child had received while in the NICU/CICU, exchange contact information, and schedule the child’s first home-based PT visit. Therapists aimed to schedule the first PT session within two weeks of hospital discharge.

**Figure 1:**
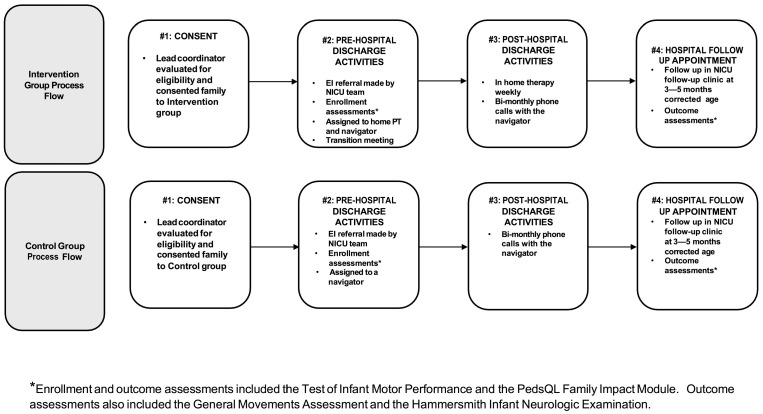
Process flow.

Study therapy was offered weekly and was provided by one of two pediatric PTs (JT, AL) with experience working in the community with complex NICU/CICU graduates at high risk for neuromotor conditions. These PTs were credentialed in pediatric rehabilitation and early childhood interventions. Weekly therapy goals were developed with parents and caregivers. Evidence-based therapeutic methods were used based on the most recent research and systematic reviews available [11,12,22]. All activities emphasized collaboration with parents as partners, active learning, and task-specific training. See Figure 1 for a detailed project process flow.

### 2.2. Outcome Measures

Infants in both groups were assessed at hospital discharge (to establish a baseline) and at 3–4 months CA to assess outcomes of the intervention. At both timepoints, infants were evaluated using the Test of Infant Motor Performance (TIMP) [23], and parents completed the PedsQL^TM^ Family Impact Module (PedsQL FIM) [24], a quality-of-life survey. All TIMP examiners were experienced pediatric therapists who had been trained by certified trainers. Reliability was regularly monitored by their respective sites. The PedsQL FIM was scored according to the manualized instructions. Additionally, at the 3–4 month follow-up visit, infants were assessed by trained and experienced examiners using the Hammersmith Infant Neurologic Exam [25] (HINE). Videos were recorded during the fidgety period for Prechtl’s General Movements Assessment (GMA) [26]. Videos were scored by certified GMA observers. Fidgety movements were assessed as normal, abnormal, sporadic, or absent. Therapists conducting the TIMP, HINE, and GMA assessments were blinded to the infants’ group assignments to the extent possible. If parents were unable to attend a clinic visit by 3–4 months CA, the study assessments could be completed at the infant’s home.

An outcome of high risk for cerebral palsy (CP) [27] was assigned if an infant met at least two-thirds of high-risk criteria as follows: moderate-severely abnormal neuroimaging findings (defined below), a HINE total score of <57 or >5 HINE asymmetries, or a GMA video showing absent, sporadic, or abnormal fidgety movements.

### 2.3. Data Collection

Medical records were reviewed to collect demographic information, birth weight, gestational age, hospital diagnoses, and readmissions. At admission to the hospital, parents provided information about their infant’s race and their home ZIP code. Neuroimaging results (cranial ultrasound or magnetic resonance imaging) were extracted from the medical record and categorized as moderate-severely abnormal if the infant had a grade 3–4 intraventricular hemorrhage, cystic periventricular leukomalacia, a brain developmental abnormality, hypoxic-ischemic encephalopathy with abnormal findings, or a neonatal stroke [5,28,29,30]. To measure neighborhood social resources, we used the Childhood Opportunity Index (version 2.0), a measure of the quality of resources and conditions in a child’s neighborhood in education, health and environment, and social and economic domains [31]. COI levels were ranked as very low, low, moderate, high, and very high opportunity neighborhoods. Nationally normed COI levels were obtained for the child’s household ZIP codes using the 2020 ZIP code data set [32].

### 2.4. Data Analysis

The primary outcome for this study was the feasibility of engagement with the study therapy, assessed as the percentage of intervention group infants seen by the study therapists within two weeks post–discharge and the percentage who received any study therapy visits over the 3–4 month study period. Secondary outcomes were compared between the intervention and standard of care groups, namely, change in TIMP and PedsQL scores from discharge to 3–4 months corrected age, results of the HINE and GMA, and engagement with each of the enrollment steps for EI. Data were summarized as numbers (percentages) and as the mean ± 1 standard deviation (SD) or as the median (25–75%). Clinical and demographic factors between groups and study outcomes were compared using *t*-tests, Chi-square tests, Fisher’s exact tests, and Mann–Whitney tests as appropriate. Statistical significance was set at a *p*-value < 0.05.

## 3. Results

**Figure 2:**
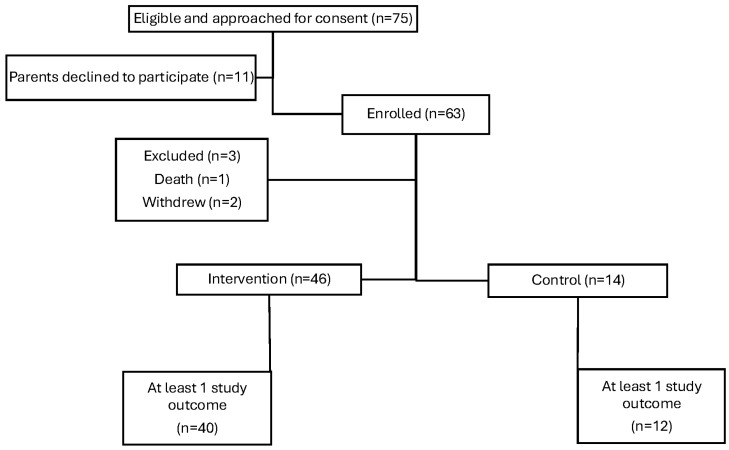
Flow of participants.

Sixty-three children were enrolled in this study across the four sites. Figure 2 shows the flow of participants through this study. Three children were excluded either due to death soon after hospital discharge (*n* = 1) or parent withdrawal from this study (*n* = 2). For the cohort overall, the study duration was 97 days (SD 22) and ended at 54.7 (SD 2.2) weeks postmenstrual age. Table 1 shows the participants’ clinical and demographic information. In the intervention group, 91.3% of the families lived in a low or very low COI ZIP code (Table 2), indicating residence in some of the most vulnerable and under-resourced neighborhoods in the United States. In contrast, in the standard of care group, 78.6% of participants lived in areas of moderate to very high COI (comparison *p* < 0.001).

**Figure 3:**
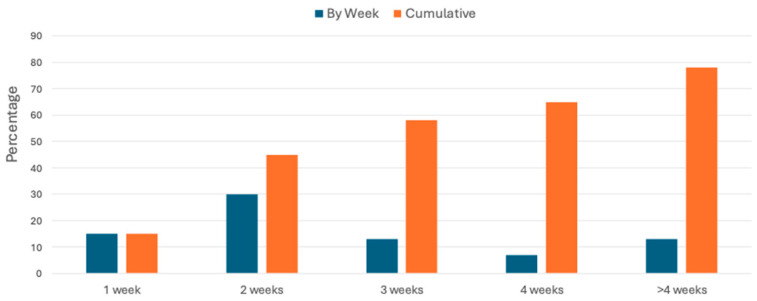
Time from discharge to first study PT visit.

Feasibility was assessed using the percentage of infants in the intervention group with a home PT visit within two weeks post-discharge and the percentage who received any study PT visits through the end of this study (Figure 3). Twenty-one infants from the intervention group (45.7%) initiated study PT within 2 weeks of hospital discharge. At least one therapy session during the study period was provided for 36 (78.2%) infants. Among the 36 infants with at least one PT visit, the initial visit was completed at a median of 12.5 days (25–75%: 9–22 days) after discharge. Days to initiation of study PT did not vary between infants with a connection call either before discharge (median 12.5, 25–75%: 7–20 days) or after discharge (median 12.5 days, 25–75%: 9–23 days, *p* = 0.55); however, three infants did not have a connection call, and none of these infants subsequently received the intervention.

In total, the INITIATE PTs completed 209 study visits in the home, with a median of 4 visits (25–75%: 1–8) per participant before the 3–4 month outcome assessment. Though the numbers were small, children from low or very low COI neighborhoods completed a similar number of therapy visits (*n* = 42, median 4.5 visits, 25–75%: 1–8) as those from moderate to very high COI neighborhoods (*n* = 4, median 3.5 visits, 25–75%: 3–7, *p* = 0.72).

Readmission to the hospital occurred in 26.7% of the families (11 infants and 1 mother). Readmission was not associated with initiation rates for study therapy (*p* = 0.18; any readmission 91.7%, no readmission 72.7%). For infants with at least 1 intervention visit, readmission was not associated with the timing of the first intervention visit, but there was a trend (*p* = 0.052) toward fewer total intervention sessions (any readmission, median 4 (25–75%: 3–7); no readmissions, median 7.5 (25–75%:5–8)).

TIMP results are shown in Table 3. Z-scores were similar between the two groups at hospital discharge and decreased similarly from discharge to 3–4 months CA for the intervention and standard of care groups. There were no differences in HINE or GMA outcomes (Table 4) at 3–4 months corrected age. At 3–4 months CA, 11 infants (7 intervention, 4 standard of care) met 2 of the 3 criteria for high risk for CP.

We noted a variable number of therapy visits before the study endpoint (0–10 visits/child) and hypothesized that infants who received therapy would improve more than infants who did not. Therefore, a post-hoc analysis was conducted to assess whether the number of therapy visits was associated with a positive change in the TIMP Score of >0.5 SD. We divided infants into terciles based on the number of therapy visits prior to the follow-up TIMP evaluation (1st: 0–1 visit, *n* = 14; 2nd: 2–7 visits, *n* = 15; and 3rd: 8–10 visits, *n* = 12). There was a significant difference in the rate of improvement >0.5 SD on the TIMP as the number of intervention sessions increased (1st tercile, *n* = 0; 2nd tercile, *n* = 2 (13%); and 3rd tercile, *n* = 5 (41%); *p* = 0.017).

Results of the PedsQL FIM are shown in Table 5. There were no differences between the groups at hospital discharge for total score, Parent HRQL Summary Score, or Family Functioning Summary Score. When assessed by tercile of number of therapy sessions, there were no significant differences between the terciles.

All infants in both groups were referred to EI at discharge by the study team. In the total cohort, 63% of parents (65.2% intervention group, 57.1% standard of care group, *p* = 0.58) reported receiving a contact from an EI coordinator by the 3–4 month study visit. EI evaluations were completed for 21 infants (35% overall; intervention group: 39.1%; standard of care group: 21.4%, *p* = 0.38). One parent declined an evaluation after learning only virtual services were available for EI. Although all infants were eligible for EI by neonatal diagnosis, after the evaluation, 2 infants did not qualify for services. Both of the infants had neonatal seizures due to hypoxic-ischemic encephalopathy or a stroke. Although the other 19 infants evaluated by EI qualified for services, just 8 had received any EI services by the end of this study at 3–4 months CA. For the entire cohort of 60 infants, the rate of receiving EI at 3–4 months of age was 13.3%, with no difference between the intervention (15.2%) and standard of care groups (7.1%, *p* = 0.44). There were no differences in the rates of EI contact or enrollment by COI level, but infants with very low to low COI zip codes were more likely to have an EI evaluation (42.2%) than infants with moderate to very high COI zip codes (13.3%, *p* = 0.042). Rates of the initial EI contact, evaluation, and enrollment by 3–4 months did not differ by readmission rates.

The navigators successfully contacted 53/60 (88.3%) parents at a median of 14 days (25–75%: 12–30 days) after discharge. On average, navigators made 3.5 (SD = 2) calls per participant during the study period to support EI enrollment. Navigators helped 18 patients to connect to outpatient therapy (intervention group, *n* = 11; standard of care group, *n* = 7). Outpatient (non-study) therapy was initiated at a median of 25 (25–75%: 14–41) days and included speech therapy and occupational therapy in addition to PT.

Barriers encountered by the study team or described by families in the process of initiating study therapy, outpatient non-study therapy, or enrollment into EI are listed in Table 6, along with solutions used during Project Initiate and recommended solutions for facilitating EI enrollment, either from parents or from the Project Initiate staff.

## 4. Discussion

The INITIATE study sought to understand and alleviate barriers in access to early community-based therapy for newborn infants at risk for neurodevelopmental impairments with public insurance transitioning from the hospital to home. This study showed that implementing prompt hospital-to-home therapy was feasible, even for families living in neighborhoods of very low or low COI. The study outcomes also suggested that infants who received consistent interventions improved their score on the motor function-based outcome measure. By establishing protocols that ensured prompt connection between the NICU/CICU and the home-based therapy, the program significantly reduced delays that hinder EI service access for infants with public insurance.

Families and hospital staff were amenable to this process. Anecdotally and supported by the high rates of recruitment (84%), families were eager to support their child’s PT transition from the hospital to home. Using the PedsQL FIM, we did not identify any changes in parent quality of life, either positive or negative, associated with study participation, indicating that prompt initiation of home PT did not negatively affect parental quality of life. Although we used only one measure of parental quality of life, the results are consistent with the few other studies on this topic that have not shown negative effects of very early intervention on parent stress and well-being [33].

Our study utilized navigators at each hospital site who functioned similarly to what has been reported in other studies [33,34,35]. Navigators supported families in many areas but with the main goal of facilitating the process for initiating EI in both groups and bridge therapy for the standard of care group. The navigator and intervention PTs were in contact throughout this study to ensure the information given to parents was clear and consistent and allowed the study team to discuss any challenges or concerns that were encountered. Our navigators were clinical coordinators or a non-treating PT, whereas in other studies, a nurse or in-home therapist served as the navigator [34,35,36]. This suggests that individuals of multiple professional backgrounds can fulfill this role.

Our findings align with other studies demonstrating the feasibility and sustainability of timely access to early therapy for infants with additional support [34,35,36,37], including the use of a family navigator. Our study PTs and navigators needed to work through several barriers to help families access the intervention (Table 6). A principal barrier was maintaining telephone contact. At the time of discharge, families are challenged with many tasks. The care needed for an infant discharged from a NICU or CICU often exceeds normal baby care and medical checkups to include subspecialty appointments, preparation of special formulas, tube feedings, medications, and/or respiratory support. Birthing parents may be still recovering from pregnancy complications themselves. High rates of stress and depressive symptoms have been reported in parents after discharge [38,39]. Given these factors, parents of newly discharged infants often do not have time or energy for “telephone tag”, particularly if they do not know who is calling. To overcome this barrier, connection calls were planned around the time of hospital discharge between the study therapist, family, and NICU/CICU staff. These calls facilitated rapport building between the family and home-based therapist, an exchange of contact information and communication preferences, and supported collaboration across teams and systems. Navigator calls were made from a recognizable hospital phone number and facilitated parent identification and return calling. Other significant barriers to providing PT sessions we noted were readmissions for illness or surgery, housing or family instability that was not identified prior to hospital discharge, and parent work schedules. These barriers could often be overcome by maintaining contact with families and flexible therapy schedules. We suggest that these barriers would also be encountered by EI systems, and some of the lessons from this study may be useful in improving the timeliness of EI enrollment.

Historically, public insurance has often been described as a barrier to EI enrollment [15,40,41]. Although our cohort all had public insurance, parents in our intervention group readily enrolled, and 78% had completed at least 1 study therapy session by the end of this study. This was possible despite over 90% of the intervention participants living in neighborhoods with low or very low child opportunity levels. We hypothesize that the structured and supportive approach contributed to this success, including the consistency of phone calls with the study navigator and the flexibility of the intervention PT for scheduling outpatient visits. We also noted that the study PTs often provided support to families beyond traditional physical therapy services, including supporting family access and awareness of community resources, including WIC, transportation services, and charities providing essential baby items. Assistance was also provided to help the family understand medical appointment schedules and provide reminders for developmental follow-up clinic appointments.

Although early intervention therapy services vary by state, we found that for our cohort, the EI system was not able to initiate therapy within 3–4 months. Only 13% of infants were receiving EI by the end of this study. Our findings are consistent with the other studies performed on this topic. For example, in Colorado, McManus et al. identified that <30% of infants referred from a safety-net hospital had ongoing EI services [14]. Nwabara et al., in Missouri, reported that even 2 years after hospital discharge, only 77% of infants referred to EI at NICU discharge had received any type of therapy [16]. The results of these studies combined with our study underscore the need for improved transition from NICU to EI services. Based on our experience in this study, the most effective method for improving access to EI would be the completion of EI enrollment processes prior to hospital discharge. This model would eliminate many of the communication barriers we observed and ensure that families receive the necessary information and support needed to connect with EI services. This would also enhance sharing of information between hospitals and EI therapists about an infant’s progress in the hospital and current goals. This recommendation warrants pilot testing, as there are likely barriers within both hospital and EI systems that are specific to local settings, and these would need to be overcome.

This study demonstrated that the dosage of early therapy may be an important influence on motor outcomes at 3–4 months of age. Though the overall change from baseline to the end of this study was not significant for the intervention group overall, children who had 8–10 PT sessions in the first 3–4 months after discharge were more likely to have a change in the TIMP Z-score of >0.5 SD. Though there is limited research on the optimal dosage of therapeutic interventions for infants at risk for developmental challenges, there is overall support in the literature that higher intensity of therapy is associated with greater improvements [13,14,40,42]. More consistent practice, when embedded in daily routines, increases the likelihood of improved outcomes, supporting the need for early, targeted therapy services during times of greatest neuroplastic potential. Eliminating therapy gaps during the transition from the hospital to home would allow for a higher intensity of services during infancy when neuroplasticity is greatest.

Strengths of this study include the inclusion of three large but diverse NICUs and one large CICU, the use of study navigators to provide additional support to families, and pediatric physical therapists with experience working with medically complex patients in under-resourced neighborhoods.

### Limitations of This Study

Although we demonstrated overall feasibility of bridge PT services at NICU/CICU discharge for infants with public insurance living in areas of very low/low COI, this pilot study had limitations that must be acknowledged and addressed in future studies. We purposely studied feasibility in infants with public insurance, as this is a group with a history of barriers to EI enrollment [14,15,16]. However, the cohort was limited to one metropolitan area (with high rates of urban poverty) and one state’s EI system, which may have biased our results, particularly for our EI enrollment outcomes. We did not find differences in access to EI services between our intervention and standard of care groups, but a study with a larger standard of care group may have shown differences, as suburban participants have different EI coordination centers, with different workflows and therapist availability. Different states in the United States have varying eligibility criteria for public insurance and interpret the EI laws differently, and our results for EI enrollment would likely vary if this study was conducted in other states. Although a limitation for our study, our findings suggest that EI systems should audit the timeliness of enrollment in infants with public insurance with medical complexity.

It also should be noted that we relied on parental reports of communication with the EI system, and these reports could not be validated with EI. It is possible that phone calls to parents by EI went to a “spam” file, or parents did not take note of the calls as they did not come from an identified EI phone number. We also did not have a way to verify if EI referrals had been received by the appropriate service office. An identified EI phone number and closed-loop communication with feedback from EI offices confirming referrals would be helpful in alleviating some of these concerns.

We did not include non-English-speaking families, as translation services for in-home intervention visits were not available. The standard of care group enrollment was smaller than anticipated. This may limit the robustness of comparative analyses between the intervention and standard of care groups. Longer follow-up of children who access early therapy is needed to better understand the impact of early therapy on motor outcomes. Missing therapy visits and outcome assessments for some of our participants are a limitation to generalization of results but also speak to the feasibility of serving an urban cohort of infants who may be medically fragile and live in areas of low and very low childhood opportunity. Therefore, we continued to try to retain infants for therapy and assessments even when there were communication gaps with families. Some of these infants may have been dropped or excluded from traditional research studies.

## 5. Conclusions

Although recent important research has focused on early detection of neuromotor impairments and the development of effective early, targeted therapy for at-risk infants, none of the benefits of these innovations can be realized if the mechanisms to provide the therapy are not in place or if these mechanisms lead to disparities in EI provision. Along with the early detection of infants at high risk for cerebral palsy, the seamless transition of rehabilitation services from the hospital to the community can have significant benefits. In this study, we have shown that early home-based therapy for medically complex children who live in underserved and under-resourced geographical areas was feasible for the majority of infants, and when consistent therapy was provided, motor function improved. These findings indicate that local and regional systemic barriers to EI enrollment should be identified and surmounted so that therapy can be implemented in early infancy, during periods of greatest neuroplasticity. Additional research with larger cohorts in more geographic locations combined with strong community advocacy will be required to bring about policy changes that ensure prompt, equitable, effective, and family-centered EI services in all communities. The success of future programs depends on the wider development of structured support systems beginning during the neonatal hospitalization and continuing into the home environment. Sharing these results with families, health and rehabilitation professionals, and state policymakers will facilitate evidence-based advocacy and a demand for increased funding for equitable systems of developmental and rehabilitative care for infants and children.

## Figures and Tables

**Table 1:** Clinical and demographic characteristics.

	Intervention Group (*n* = 46)	Standard of Care Group (*n* = 14)
Male, N (%)	20 (43.4%)	9 (64.3%)
Race, N (%)		
Black	26 (56.6%)	4 (28.6%)
Other	10 (21.7%)	4 (28.6%)
Other/White	0	1 (7.1%)
White	10 (21.7)	4 (28.6%)
Undisclosed	0	1 (7.1%)
Ethnicity, N (%)		
Hispanic or Latino	18 (39.1%)	9 (64.3%)
Non-Hispanic or Latino	28 (60.9%)	5 (35.7%)
Birth weight (grams), mean (SD)	2093 (1051)	2540 (1196)
<1000 g, N (%)	13 (28.3%)	3 (21.4%)
Gestational age (weeks), mean (SD)	33.9 (5.2)	35 (6.2)
Neuroimaging: moderate-severe abnormal findingsm N (%)	15 (32.6%)	8 (57.1%)
Congenital anomalies/syndrome, N (%)	19 (41.3%)	6 (42.8%)
Congenital heart disease, N (%)	7 (15.5%)	3 (21.4%)
Neonatal surgery, N (%)	9 (19.5%)	4 (28.4%)
Technology dependent at discharge, N (%)		
Tube feedings, N (%)	4 (8.7%)	1 (7.1%)
Supplemental oxygen, N (%)	5 (10.9%)	0

Data are presented as the N (%) or mean (SD). No significant differences between any clinical or demographic characteristics.

**Table 2:** Childhood opportunity index.

	Intervention Group	Standard of Care Group	*p*-Value
Very low, N (%)	34 (79.3%)	1 (7.1%)	<0.001
Low, N (%)	8 (17.4%)	2 (14.3%)	
Moderate, N (%)	3 (6.5%)	2 (14.3%)	
High, N (%)	1 (2.2%)	5 (35.7%)	
Very high N (%)	0	4 (28.6%)	

**Table 3:** TIMP results.

TIMP Results	Intervention Group (*n* = 46)	Standard of Care Group (*n* = 14)	*p*-Value
Discharge TIMP assessed, N (%)	44 (95.7%)	12 (85.7%)	0.19
Age at discharge TIMP (weeks PMA), mean (SD)	40.45 (2.75)	42.9 (1.78)	0.005
Baseline TIMP Z-score, mean (SD)	−0.28 (0.74)	−0.79 (0.45)	0.03
3–4 month TIMP assessed, N (%)	36 (78.3%)	10 (71.4%) *	0.6
Age at 3–4 month TIMP (weeks PMA), mean (SD)	53.4 (6.4)	54.8 (2.5)	0.47
3–4 month TIMP Z-score, mean (SD)	−0.8 (1.1)	−1.23 (1.1)	0.27
3–4 month TIMP Score below or far below average range, N (%)	10 (27%)	5 (50%)	0.17
Paired discharge and 3–4 month TIMP Scores, N (%)	36 (78.3%)	10 (71.4%)	0.6
Change Z-scores from discharge to 3 months **	−0.47 (1.2)	−0.42 (1.2)	0.91

* One additional child was assessed with the TIMP at 60 weeks PMA—no score was obtained. ** N = those with paired TIMP Scores from discharge to 3–4 months CA.

**Table 4:** HINE and GMA outcomes.

Assessment	Intervention Group (*n* = 46)	Standard of Care Group (*n* = 14)	*p*-Value
Number with HINE (%)	34 (74%)	10 (71.4%)	0.85
HINE total score, mean (SD)	61.3 (7.7)	57.6 (10.1)	0.21
HINE score < 57, N (%)	9 (26%)	5 (50%)	0.16
HINE asymmetries > 5, N (%)	2 (5.9%)	0	0.43
HINE asymmetry score, mean (SD)	1.7 (1.9)	2 (0.9)	0.62
Number with GMA (%) *	33 (71.7%)	12 (85.7%)	0.29
GMA-atypical ** fidgety movements, N (%)	4 (12.1)	2 (16.7%)	0.69
High risk for CP, N (%) ***	7 (15.2%)	4 (28.6%)	0.26

* One child with FM classification only. ** Absent, sporadic, or abnormal fidgety movements. *** Defined as at least 2 of the following: moderate-severely abnormal neuroimaging, HINE score (<57 or >5 asymmetries), and/or atypical fidgety movements.

**Table 5:** PedsQL FIM Scores.

PEDS QL FIM Scores	Intervention Group (*n* = 46)	Standard of Care Group (*n* = 14)	*p*-Value
Completed predischarge PedsQL, N (%)	38 (91.3%) *	14 (100%)	0.23
Postmenstrual age at completion	39.8 (2.6)	41.3 (2.2)	0.06
Predischarge FIM Total Score, mean (SD)	83.6 (15.6)	75.3 (17.6)	0.1
Predischarge FIM Parent HRQL Summary Score, mean (SD)	84.3 (17.1)	76.3 (19.7)	0.15
Predischarge FIM Family Functioning Score, mean (SD)	84.4 (17.7)	76.1 (18.5)	0.14
Completed follow-up PedsQL, N (%)	30 (65%) *	11 (78.6%)	0.52
Postmenstrual age at completion	54.3 (5.0)	55 (2.4)	0.64
Follow up FIM Total Score, mean (SD)	86.4 (14.6)	78.2 (19.9)	0.16
Follow up FIM Parent HRQL Summary Score, mean (SD)	85.2 (17.2)	78.1 (18.3)	0.26
Follow up FIM Family Functioning Score, mean (SD)	86.8 (17.8)	76.7 (28.5)	0.19

* One incomplete form at each age.

**Table 6:** Barriers identified to providing study therapy, outpatient appointments, and EI enrollment with possible solutions.

**Barriers to Providing Study Therapy Visits**	**Solutions Used in Project Initiate**
Communication lacking between the NICU/CICU and study therapists	Closed-loop communication for referrals Set up therapy appointments before discharge
Communication difficulties between the study therapist and parent	Before discharge, establish a reliable communication plan (via phone or email, correct phone number, best times for phone calls)
Housing or family instability	Refer back to NICU/CICU social worker
Infant readmission to the hospital Parental illness or surgery	Continue contact with families during hospitalization
Parent work schedule	Flexible hours for therapy sessions
**Barriers to Navigating Outpatient “Bridge” Services**	**Solutions Used in Project Initiate**
Communication delays between navigator and parent	Telephone calls made from an identified phone number so parents know who is calling, use of text messaging, or email
Lack of NICU clinician referral for outpatient therapy at discharge	Navigator contacted physicians for referrals
Lack of local options or long waiting lists for outpatient pediatric therapy services (including lack of Medicaid providers)	Navigator researched options for parents Recommendation of online resources (e.g., https://pathways.org, accessed on 11 December 2024)
Parent work schedule	Navigator researched providers with extended hours
Transportation difficulties	Navigator provided information about transportation resources available within the community
Infant readmission to hospital	Navigator encouraged parents to request therapy during hospitalization Continue contact with families during hospitalization
**Barriers to Enrollment in EI**	**Recommended Solutions**
Uncertainty about whether referral was received by EI	After discharge, EI should provide a notification that a referral has been received IFSP should be made before discharge
Communication between the parent and EI	Telephone calls should be made from an identified phone number, so parents know who is calling, or allow contact by email
Video services only offered	Increasing numbers of therapists offering EI to provide more choice
Confusion about qualifying diagnoses	Education of hospital staff, EI coordinators, EI providers, and families about eligible conditions. Develop an IFSP before discharge in collaboration with the family, the EI team, and the NICU team
Difficulties scheduling in-home evaluations	Schedule the first therapy appointment with parents before hospital discharge
Shortage of therapists in EI	Advocacy and improving the training “pipeline” for therapists—develop a statewide task force with professional schools Use outpatient therapy services as a “bridge” to EI
Infant readmission to the hospital	EI coordinators should continue contact with family during admission and schedule appointments for home therapy promptly after discharge

## Data Availability

Research data are not available due to privacy considerations.
